# Alpha-1-antitrypsin interacts with gp41 to block HIV-1 entry into CD4+ T lymphocytes

**DOI:** 10.1186/s12866-016-0751-2

**Published:** 2016-07-29

**Authors:** Xueyuan Zhou, Zhu Liu, Jun Zhang, Joseph W. Adelsberger, Jun Yang, Gregory F. Burton

**Affiliations:** 1 Clinic Services Program, Leidos Biomedical Research Inc., Frederick National Laboratory for Cancer Research, Frederick, MD 21702 USA; 2 Hainan Key Laboratory for Sustainable Utilization of Tropical Bioresources, College of Agriculture, Hainan University, Haikou, Hainan 570228 China; 3 Department of Chemistry, University of Alabama at Birmingham, Birmingham, AL 35294 USA; 4 Department of Chemistry and Biochemistry, Brigham Young University, Provo, UT 84602 USA

**Keywords:** HIV-1, Alpha-1-antitrypsin, Reverse transcriptase, Integrase, gp41

## Abstract

**Background:**

Study of a clinic case reveals that alpha-1-antitrypsin (AAT) deficiency is related to CD4+ T cell count decline and AIDS progression, suggesting that AAT might be an endogenous inhibitor of HIV/AIDS. Previous study shows that AAT inhibits HIV-1 replication in infected host cells and the C-terminus fragment of AAT, VIRIP, interferes with HIV-1 infection. However, it is still unclear whether and how intact AAT inhibits HIV-1 infection. It is also unknown what the mechanism of AAT is and which critical step(s) are involved.

**Results:**

In the present study, the C-terminus of AAT (C) was synthesized. C terminus-truncated AAT (ΔAAT) was also prepared by digesting AAT with metalloproteinase. Primary CD4+ T cells were then co-cultured with HIV-1 with the presence or absence of AAT/C/ΔAAT to detect *cis*-infection of HIV-1. The interaction between AAT/C/ΔAAT and gp120/gp41 was also measured. Meanwhile, HIV-1 reverse transcriptase activity and viral DNA integration were also detected in these lymphocytes. The results demonstrated that AAT and C, not ΔAAT, inhibited HIV-1 entry by directly interacting with gp41. Meanwhile, AAT, C and ΔAAT could not directly interfere with the steps of viral RNA reverse transcription and viral DNA integration.

**Conclusion:**

AAT inhibits HIV-1 entry by directly interacting with gp41 through its C-terminus and thereby inhibits HIV-1 infection.

**Electronic supplementary material:**

The online version of this article (doi:10.1186/s12866-016-0751-2) contains supplementary material, which is available to authorized users.

## Background

Human immunodeficiency virus type 1 (HIV-1) is hard to propagate in infected and unaltered whole blood, unless the blood is diluted and active lymphocytes are present [1, 2]. Investigations of HIV-1 replication in patients reveal that HIV-1 proliferation is confined in the lymph nodes where the concentration of specific serum constituents is lower [3, 4]. These facts suggest that human body produces endogenous inhibitors for HIV and acquired immune deficiency syndrome (AIDS) progression. Further studies show that various components in human blood and tissues are possible candidates [5–7]. Among these components, serine protease inhibitors are promising. Studies report that salivary secretary leukocyte protease inhibitor suppresses HIV-1 replication [8, 9]. Researchers also focus on alpha-1-antitrypsin (AAT), a 394 amino acid, 52 kDa glycoprotein synthesized in the liver and secreted into the circulation [10–15]. AAT is consistently present in the serum of healthy individuals (1.5 ~ 3.5 mg/mL), although its concentration can increase several times upon inflammation [16, 17]. Study of a clinic case reveals that pre-existing AAT deficiency is associated with accelerated HIV/AIDS progression, which suggests that AAT might be an endogenous HIV/AIDS suppressor [13, 18].

The lifespan of HIV can be divided into two major processes, infection and replication [19, 20]. HIV-1 infection begins with the interaction between viral gp120/gp41 and CD4/co-receptors on host cells and ends with the integration of viral DNA into host genome. This process contains several middle steps, including the entry of viral core, reverse transcription of viral RNA and nuclear translocation of viral DNA [21, 22]. HIV-1 replication begins with the transcription and translation of viral genes, which lead to the package, budding and release of new virus, which is then processed to become infection-competent virus [23]. Our previous study reveals that AAT enters the cytosol of infected CD4+ T cells through LRP1-mediated endocytosis process, where it directly interacts with nuclear factor kB (NF-kB) inhibitor (IkBα) and thereby alters its ubiquitinylation pattern to block NF-kB activation and HIV-1 replication [14, 24, 25]. Munch’s study suggests that the C-proximal fragment of AAT (A.A. 353–372), VIRIP, inhibits HIV-1 infection [12]. However, it is unclear whether and how intact 55KD AAT interacts with gp120-covered gp41on the viral membrane to inhibit HIV-1 infection, due to its big size that might shield its VIRIP domain. It is also unknown whether AAT inhibits HIV-1 infection by targeting on single or multiple steps: viral core entry, viral RNA reverse transcription or viral DNA integration. Moreover, it is still unclear whether C-terminus of AAT is the only functional domain.

In the present study, we sought to investigate the accurate mechanism of intact AAT inhibiting HIV infection. The results demonstrate that AAT and synthesized C-terminus fragment of AAT (C), not C-terminus-truncated AAT (ΔAAT), inhibits HIV-1 entry into CD4+ T cells. The inhibitory effect is mediated through their direct interactions with gp41. Additionally, AAT, C and ΔAAT did not directly affect the steps of viral RNA reverse transcription and viral DNA integration. Thus, our results clarify how AAT inhibits HIV-1 infection in primary CD4+ T cells. Combined with the previous finding that AAT inhibits HIV-1 replication in infected cells [15, 24, 25], it is clear that AAT can suppress HIV/AIDS pathogenesis through inhibiting both HIV infection and replication in vitro, which might provide some useful information for HIV/AIDS study and drug development.

## Methods

### Reagents

Human plasma AAT was obtained from Sigma [purity and quality were analyzed by electrophoresis and mass spectrometry following the methods described below (Additional file 1: Figure S1)]. ΔAAT were prepared by digesting AAT. Briefly, AAT and metalloproteinase from *S. aureus* (Sigma) were co-cultured at 40:1 (molar ratio) for 3 h at 37 °C in 50 mM NH_4_HCO_3_. Next, protease was removed by using HiTrap benzamidine column (Amersham) and then passed through 10KD MWCO spin filter (Millipore) to separate truncated AAT. The truncation of AAT was verified by electrophoresis and mass spectrometry (Additional file 1: Figure S1) and also justified by the loss of ability to bind trypsin and elastase (Sigma) [26, 27]. C-terminus of AAT (C) (A.A. 345–384: KGTEAAGAMFLEAIPMSIPPEVKFNKPFVFLMIDQNTKSP) was synthesized in Genscript and the purity and accuracy of peptide was also analyzed by electrophoresis and mass spectrometry (Additional file 1: Figure S1). HIV-1 integrase inhibitor raltegravir (RAL), HIV-1 reverse transcriptase inhibitor emtricitabine (FTC) and HIV-1 entry inhibitor enfuvirtide (ENF) were from Santa Cruz Biotechnology.

### HIV-1 preparation

HIV-1 stock was prepared by propagating HIV-1_NL4.3_ (X4 virus) in mitogen-stimulated peripheral blood mononuclear cells (PBMC) as before [24]. Unless otherwise noted, HIV-1 infection was performed using HIV-1_NL4.3_. HIV-1 primary isolates, 91US054 (X4 virus) or 92US714 (R5 virus) (NIH AIDS Research and Reference Reagent Program), were also propagated in mitogen-stimulated PBMCs in complete culture medium (CM) [RPMI1640 with HEPES (20 mM), nonessential amino acids, L-glutamine (2 mM), 10 % heat-inactivated, defined FBS (Life Technologies/Invitrogen) and gentamicin (50 mg/ml; Life Technologies)]. Additionally, GFP-labeled HIV-1_NL4.3_ was made by transfecting a vector coding for HIV-1_NL4.3_ with GFP insertion in gag region (HIV-1 Gag-iGFP) into HEK293T cells. GFP-labeled pseudotyped HIV-1_NL4.3_ was made by transfecting a vector coding for HIV-1_NL4.3_ with GFP insertion in gag region and VSV-G replacing of Env (VSV-G-pseudotyped HIV-1 Gag-iGFP) into HEK293T cells (both vectors were generous gifts from Dr. Yuntao Wu; Dr. Wu also provided us pure gp120 and gp41). After harvesting, virus was purified, concentrated, and stored in −80 °C.

### CD4+ T cell isolation and infection

PBMCs were extracted from the whole blood of healthy, HIV-negative donors using Ficoll-Paque-Plus (GE Healthcare) as directed. CD4+ T cells were then isolated from these PBMCs using a CD4+ T cell isolation Kit II (Miltenyi Biotech) as directed. Next, isolated CD4+ T cells were activated and maintained as before [15]. To ensure that CD4- and coreceptor-dependent infection (cis-infection) is not interfered by the endocytosis of viral particle that is an alternative way of HIV infection in some cases (trans-infection) [28], activated CD4+ T cells were cultured for 30 min in conditioned complete medium [complete medium with the endocytosis inhibitor cocktail (5 μg/mL methyl-beta-cyclodextrin, filipin and chlorpromazine)] before and during infection, which was washed off after 2 h’ infection and maintained in normal complete medium when prolonged incubation was needed.

### ELISA assay for HIV-1 p24 detection

To detect cytosol p24, CD4+ T cells were cultured with HIV-1. These cells were then suspended in Lysis Buffer [50 mM Tris–HCl (pH7.4), 1 % CHAPS, 250 mM NaCl, 0.5 % Triton X-100, 1 % Igepal CA-630, 1 mM DTT, 1 mM Na_3_VO_4_, 1 mM NaF, 1 mM PMSF, 4 mM EDTA, protease inhibitor cocktail (Roche)] and vortexing for 60 s. The mixture was then incubated on ice for 15 min and homogenized with a small gauge needle by drawing 3 times. After homogenizing, the mixture was centrifuged at 14,000 × g for 10 min at 4 °C to collect the supernatant (whole cell proteins containing viral proteins). HIV-1 p24 was detected using the HIV-1 p24 antigen ELISA kit (ZeptoMetrix Corporation) following the direction. For HIV-1 replication, the supernatant fluid of the culture system was collected to detect HIV-1 p24 using the HIV-1 p24 antigen ELISA kit (ZeptoMetrix Corporation) following the direction.

### HIV-1 RNA detection

Supernatant with HIV-1 viral particles was collected for viral RNA quantitation. Viral RNA was isolated using QIAamp viral RNA mini Kit (Qiagen). Isolated viral RNA was reverse-transcribed into cDNA using random primers and Superscript III reverse transcriptase (Invitrogen). Quantitative RT-PCR using TaqMan Universal PCR Master Mix (Applied Biosystems) used the following primers and probe: forward primer: 59-TGGGTACCAGCACACAAAGG-39 (nt 3696 in HXB2); reverse primer: 59-ATCACTAGCCATTGCTCTCCAAT-39 (nt 3850 in HXB2); and probe: ATTGGAGGAAATGAAC-MBG (FAM labeled) at 900 nM (primers) and 250 nM (probe). Quantitative PCR conditions were as follows: 50 °C for 2 min (1 cycle), 95 °C for 10 min (1 cycle), followed by 60 cycles of: 95 °C for 15 s and 60 °C for 1 min in an ABI 7500 thermocycler (Applied Biosystems). A standard curve was prepared using known concentrations (i.e., copy numbers) of ACH-2 DNA to determine the number of copies of viral RNA present in the cultures.

### Alu-PCR assay for viral DNA integration detection

Cells were washed and resuspended in DNA-STAT 60 (Tel-Test) to extract genomic DNA following the protocol manufacture provided. Extracted DNA was then used to run Alu-gag PCR [forward primer: 5′-GCC TCC CAA AGT GCT GGG ATT ACA G-3′; reverse primer (HIV gag; nt 1505–1486): 5′-GTT CCT GCT ATG TCA CTT CC-3′]. PCR conditions were as follows: 95 °C × 2 min (1 ×), followed by 40 cycles of: 95 °C × 15 s, 50 °C × 15 s, 72 °C × 3.5 min. After Alu-gag PCR, RU5 quantitative real time PCR was carried out with the following primers and probe. R forward primer (nt 518–539): 5′-TTA AGC CTC AAT AAA GCT TGC C-3′, U5 reverse primer (nt 647–628): 5′-GTT CGG GCG CCA CTG CTA GA-3′, RU5 probe (nt 584–559): 5′-FAM-CCA GAG TCA CAC AAC AGA CGG GCA CA-MBG-3′) as directed. Meanwhile, beta-globin was also detected using the same DNA as template. Forward primer: 5′- CCC TTG GAC CCA GAG GTT CT-3′, reverse primer: 5′- CGA GCA CTT TCT TGC CAT GA-3′, probe: 5′- VIC-GCG AGC ATC TGT CCA CTC CTG ATG CTG TTA TGG GCG CTC GC-TARAMA-3′) as directed. PCR conditions were as follows: 50 °C × 2 min (1 ×), 95 °C × 20 s (1 ×); followed by 60 cycles of: 95 °C × 3 s, 60 °C × 30 s. A standard curve was prepared using known concentrations (i.e., copy numbers) of ACH-2 DNA to determine the number of copies of viral DNA integrated into the host genome. The primers were used at 900 nM and the probe at 250 nM.

### Detection of HIV-1 reverse transcriptase activity

Infected CD4+ T cells were suspended lysis buffer [50 mM Tris (pH 7.4), 500 mM NaCl, 1 % Triton X-100, 2 mM PMSF, 20 % glycerol, 1 mM DTT and protease inhibitor cocktail (Roche)] and vortexed for 60 s. The mixture was incubated on ice for 45 min and homogenized with a small gauge needle by drawing 3 times. After homogenizing, the mixture was centrifuged at 14,000 × g for 10 min at 4 °C to collect the supernatant containing HIV-1 reverse transcriptase. Next, the supernatant was treated with DNase I (Ambion, Inc.) to remove contaminating DNA. The activity of HIV-1 reverse transcriptase was detected by EnzChek® Reverse Transcriptase Assay Kit (Invitrogen) following the protocol manufacture provided.

### Protein extraction

For whole cell extraction, cells were collected and washed. For viral protein extraction, HIV-1 particles were collected, washed, and concentrated by centrifuging at 100,000 × g for 2 h at 4 °C. RIPA Lysis Buffer was then added to cell or viral pellet and vortexed for 60 s. The mixture was incubated on ice for 45 min and homogenized with a small gauge needle by drawing 3 times. After homogenizing, the mixture was centrifuged at 14,000 × g for 10 min at 4 °C to collect the supernatant (whole cell proteins or viral proteins).

For membrane protein extraction, cells were washed with cold PBS and membrane proteins were extracted with membrane protein extraction kit (BioVision) following the protocol manufacture provided.

### Western blot assay

Western blotting was carried out following the previous protocol [29]. After blocking, the membrane was incubated with the primary antibody [goat anti-human gp120 (Abcam), goat anti-human gp41 (Abcam), goat anti-human AAT (Bethyl), mouse anti-human CD4 (Immunotech), mouse anti-human CCR5 (Pharmingem), mouse anti-human CXCR4 (R&D), or rabbit anti-human β-actin (Abcam)]. Next, secondary antibody [anti-goat IgG-HRP (Chemicon international), anti-mouse IgG-HRP (Chemicon international), or anti-rabbit IgG-HRP (Chemicon international)] was used to link to the primary antibody and then developed using ECL Advance Western Blotting Detection Kit (GE Healthcare).

### Immunoprecipitation assay

Extracted proteins were used to carry out immunoprecipitation assay following our previous protocol with a pre-clearing step [24, 25].

### Flow cytometry assay

Antibodies used were: CD4 allophycocyanin (APC), CXCR4 pacific blue, CCR5 fluorescein isothiocyanate (FITC) and antibody isotope controls were from BD Biosciences. Upon analysis, cells were washed and incubated for 20 min at room temperature in PBS containing 2 % BSA and antibodies or antibody isotope controls. Cells were then collected and washed twice in ice-cold PBS. The samples were analyzed on flow cytometer. For CD4+ T cell infection with HIV-1_NL4.3_ (HIV-1 Gag-iGFP), activated CD4+ T cells were infected with GFP-labeled HIV-1_NL4.3_ and unbound viruses were then removed by washing three times. GFP in CD4+ T cells was analyzed on flow cytometer to determine HIV-1 entry.

### Membrane receptor interaction identification

For HIV-1 membrane receptor identification, HIV-1 was washed and concentrated by centrifuging at 100,000 × g for 2 h at 4 °C. Next, viral particles were incubated with AAT and dithiobis (succinimidylpropionate) (DSP, Thermo Scientific) was added to stabilize the interaction between AAT and HIV-1 membrane protein. Next, the reaction was stopped by adding Tris buffer and viral proteins were then extracted following the whole viral protein extraction protocol described above. For cell membrane receptor identification, cells were incubated with AAT. Excess AAT was washed off and DSP was added to stabilize the interaction between AAT and cellular membrane protein. The reaction was also stopped by adding Tris buffer and the membrane proteins were extracted with membrane protein extraction kit (BioVision) following the provided protocol described above. Isolated cell membrane proteins or viral membrane proteins were incubated with AAT-specific antibody to precipitate proteins that interacted with AAT. Precipitated proteins were separated by SDS-PAGE and specific protein bands were cut and digested with a sequencing grade modified trypsin (Promega) to identify the proteins by peptide mass fingerprinting assay using a high-performance liquid chromatography-mass spectrometry system (HPLC-MS) following the protocol described below.

### In-gel digestion for peptide mass fingerprinting assay

The proteins were separated by SDS-PAGE and the appropriate bands were cut for peptide mass fingerprinting assay following our previous protocol [24]. Digested peptides were analyzed on the HPLC-MS system (Applied Biosystem API QSTAR pulsar I LC/MS system or Thermo scientific LTQ XL Orbitrap LC/MS system). The proteins were identified by searching the specific mass spectrum in the database (Mascot).

### In-gel filter*-*aided sample preparation (FASP) for peptide mass fingerprinting assay

Proteins were separated by SDS-PAGE and the appropriate bands were collected for FASP assay following the previous protocol [24]. The peptides were also analyzed on Applied Biosystem API QSTAR pulsar I LC/MS system. The proteins were identified by searching the specific mass spectrum in the Mascot database.

### Surface plasmon resonance (SPR) assay

AAT, ΔAAT or C-terminus fragment of AAT binding with gp41 and gp120 was detected on a BIAcore 3000 biosensor system (Pharmacia Biosensor AB) using SPR assay. Briefly, a carboxymethylated CM5 sensor chip was activated with 1:1 mixture of 0.4 M N-ethyl-N -(3-dimethylaminopropyl) carbodiimide and 0.1 M′ N-hydroxysuccinimide. AAT (0.5 g/L in 10 mM NaOAc, pH5), ΔAAT (0.47 g/L in 10 mM NaOAc, pH5) or synthesized C-terminus fragment of AAT (0.05 g/L in 10 mM NaOAc, pH5) were then immobilized on the sensor chip by amine-coupling according to the manufacturer’s instructions. Unreacted sites were blocked with 1 M ethanolamine/HCl (pH 8). Control flow cells (blank) were activated and blocked in the absence of AAT, ΔAAT or synthesized C-terminus fragment of AAT. Flow cells were routinely equilibrated with running buffer (PBS, 0.005 % surfactant P20). Analyst gp41 and gp120 were diluted in the running buffer and allowed to interact with the sensor surface by a 250-s injection. Different concentrations of gp120 and gp41 were injected, each at the flow rate of 10 μL/min at 25 °C. Data from duplicate assays were modeled for binding equilibrium.

### Statistical analysis

Every experiment was repeated at least three times with different donors. Analysis of data was performed using Student *t* test. *P* values of ≤ 0.05 were considered significant. Unless specifically stated, the error bars indicate the standard errors of the means (SEM).

### Studies using human cells/tissues

Studies using blood or tissue-derived cells obtained from humans were reviewed and approved by an appropriate institutional review committee.

## Results

**Fig. 1:**
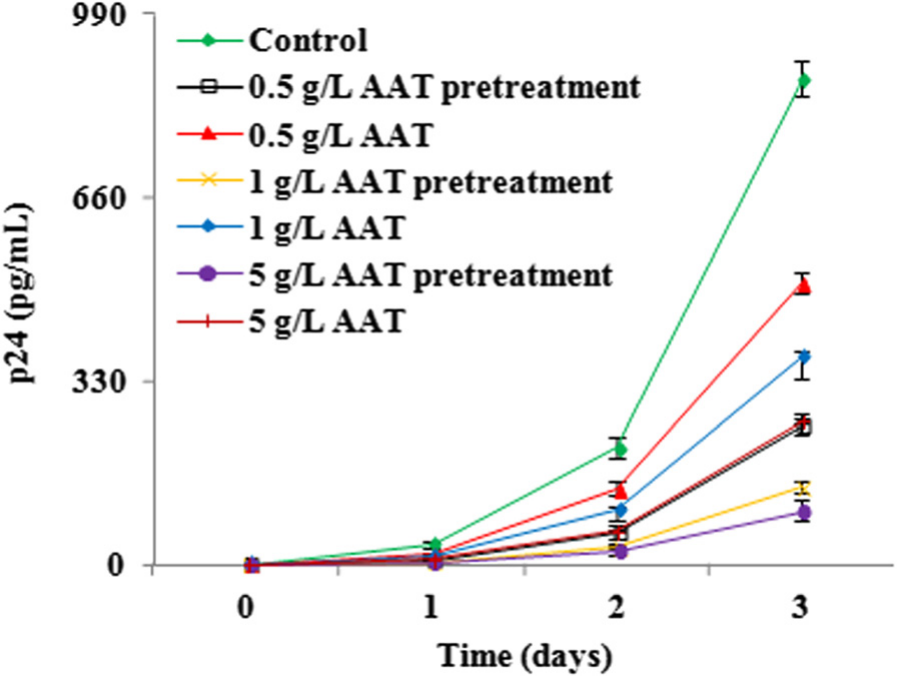
AAT inhibited HIV-1 replication. Activated primary CD4+ T cells were divided into two aliquots. One aliquot was treated with AAT (0.5, 1, or 5 g/L) for 1 h and then infected with HIV-1_NL4.3_ for 2 more hours without removing AAT. After infection, unbound viruses were removed by washing the T lymphocytes for three times and cells were then cultured with AAT as before infection. The other aliquot was directly infected with HIV-1_NL4.3_ for 2 h and unbound viruses were then removed by washing three times. Infected T cells were then treated with the presence or absence of AAT. HIV-1 production was detected by measuring HIV-1 p24 (in the supernatant) at different time point in each group

Study shows that the C-proximal fragment of AAT (A.A.: 353–372), VIRIP, inhibits HIV-1 infection [12]. However, it is still unclear whether and how intact AAT inhibits HIV-1 infection and whether VIRIP is the only functional domain. It is also unknown whether AAT inhibits HIV-1 infection by targeting virus entry, viral RNA reverse transcription or viral DNA integration. To address these issues, activated primary CD4+ T cells were pretreated with AAT at different concentrations and then infected with HIV-1_NL4.3_ without removing AAT in the conditioned complete medium containing an endocytosis inhibitor cocktail to get rid of the possible trans-infection of HIV-1 [28]. After infection, cells were washed and incubated with AAT again (same condition as before infection) to detect HIV-1 replication. The results demonstrated that CD4+ T cells with AAT pretreatment produced much less virus than those without AAT pretreatment. Meanwhile, both of these cells produced less virus than lymphocytes without AAT treatment (Fig. 1). These results suggest that, besides inhibiting HIV-1 replication in infected host cells, intact AAT might also interfere with HIV-1 infection of uninfected cells. However, it is still unclear which step(s) AAT targets to exert its inhibitory effect on HIV-1 infection.

**Fig. 2:**
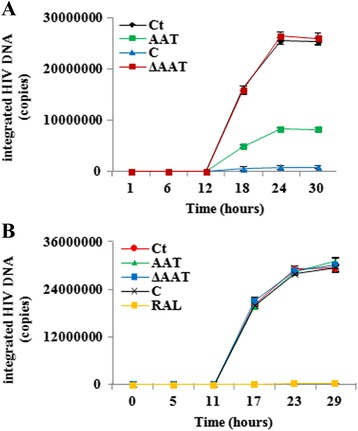
AAT, C and ΔAAT did not directly target on viral DNA integration. **a** CD4+ T cells were pretreated in the presence or absence of AAT/C/ΔAAT and then infected by HIV-1_NL4.3_ without removing the reagents. Next, cells were washed to remove unbound viruses and reagents and then incubated for 1, 6, 12, 18, 24, or 30 h in the presence or absence of AAT/C/ΔAAT (the same condition as before infection) to isolate genomic DNA. Viral DNA integration was detected by Alu-PCR. **b** CD4+ T cells were also infected with HIV-1_NL4.3_ without AAT/C/ΔAAT pretreatment. Next, cells were incubated in the presence or absence of AAT, C, ΔAAT or HIV-1 integrase inhibitor raltegravir (10^−4^ g/L). After 0, 5, 11, 17, 23, or 29 h incubation, DNA was extracted from these CD4+ T cells to detect viral DNA integration. Genomic beta-globin was also detected as the endogenous control. Ct: control group without reagent treatment; RAL: raltegravir treatment

To determine the mechanism of AAT's inhibition, we first investigated whether AAT inhibited HIV-1 infection by blocking viral DNA integration, which is the closest step to HIV-1 replication. CD4+ T cells were therefore incubated in the presence or absence of AAT/ΔAAT/C and then infected with HIV-1_NL4.3_ without removing the reagents. Because 0.5–5 g/L AAT inhibits HIV-1 infection without affecting the viability of CD4+ T cells and this concentration is within the range found in the human body (Fig. 1 and Additional file 2: Figure S2) [15, 24], 0.5 g/L AAT was used in the remainder of the study. Moreover, 0.5 mg/mL AAT also had no obvious effect on inducing the precipitation of HIV-1_NL4.3_ particles (Additional file 3: Figure S3). Meanwhile, relative equivalent amounts of ΔAAT (0.47 g/L) and C (0.05 g/L) were also included to detect their effect on viral DNA integration (Fig. 2). The results demonstrated that CD4+ T cells with AAT pre-treatment had less integrated viral DNA (>60 % decrease; *p* < 0.01). Meanwhile, C-pretreated CD4+ T cells also had less integrated viral DNA than AAT-pretreated CD4+ T cells (>70 % decrease; *p* < 0.001) (Fig. 2a). If CD4+ T cells were infected with HIV-1_NL4.3_ at first and then co-cultured with AAT, C, ΔAAT or HIV-1 integrase inhibitor raltegravir, the results showed that AAT, C and ΔAAT had no obvious effect on viral DNA integration while positive control raltegravir almost completely blocked viral DNA integration without altering the cell viability (>98 % decrease; *p* < 0.001) (Fig. 2b and Additional file 2: Figure S2). Additionally, the effect of AAT, C and ΔAAT on viral DNA integration was also tested on different tropism of primary HIV-1 isolates, HIV-1_92US714_ and HIV-1_91US054_. The results demonstrated that AAT, C and ΔAAT also had similar effect on these HIV-1 isolates (Additional file 4: Figure S4). Thus, these results suggested that AAT and C, not ΔAAT, could inhibit HIV-1 infection of CD4+ T cells. However, the inhibition was not mediated though directly blocking viral DNA integration.

**Fig. 3:**
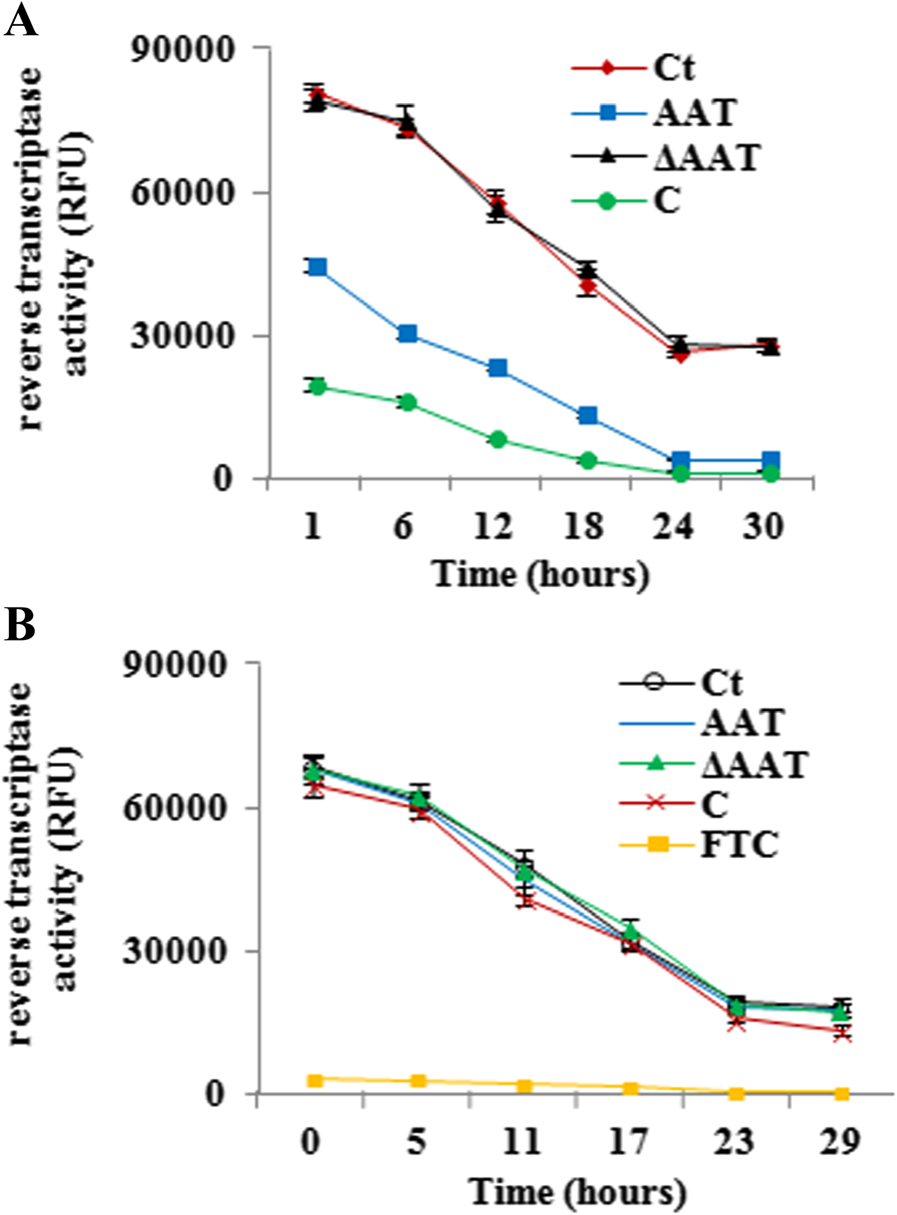
AAT, C and ΔAAT did not directly target viral RNA reverse transcription. **a** CD4+ T cells were pretreated with or without AAT/C/ΔAAT and then infected by HIV-1_NL4.3_ without removing the reagents. Infected CD4+ T cells were then washed to remove unbound viruses and reagents and then incubated for 1, 6, 12, 18, 24, or 30 h in the presence or absence of AAT/C/ΔAAT (the same condition as before infection) to isolate whole cell proteins with viral proteins. The activity of HIV-1 reverse transcriptase in normalized whole extracts was detected following the protocol described in the Materials and Method. **b** CD4+ T cells were also infected with HIV-1_NL4.3_ without pretreatment and then incubated with the presence or absence of AAT, C, ΔAAT or HIV-1 reverse transcriptase inhibitor emtricitabine (10^−3^ g/L). After 0, 5, 11, 17, 23, or 29 h’ incubation, the whole cell proteins with viral proteins were extracted from these CD4+ T cells and normalized to detect the activity of HIV-1 reverse transcriptase. Ct: control group without reagent treatment; FTC: emtricitabine treatment

Next, we sought to clarify whether AAT blocked HIV-1 infection by directly interfering with viral RNA reverse transcription. CD4+ T cells were therefore treated with AAT/C/ΔAAT and then infected by HIV-1_NL4.3_ without removing the reagents. The activity of HIV reverse transcriptase was then measured in CD4+ T cell lysates (Fig. 3). The results showed that the activity of HIV-1 reverse transcriptase in AAT-pretreated CD4+ T cells was lower than that in untreated CD4+ T cells (>50 % decrease; *p* < 0.05), which could be caused by the lower amount of HIV-1 reverse transcriptase entry into the host cells (Fig. 3a). Meanwhile, the activity of HIV reverse transcriptase from C-pretreated CD4+ T cells was also lower than that from AAT-pretreated CD4+ T cells (>50 % decrease; *p* < 0.005) (Fig. 3a). When CD4+ T cells were infected first and then cultured with AAT, C, ΔAAT or HIV-1 reverse transcriptase inhibitor emtricitabine, the results demonstrated that AAT, C and ΔAAT had no obvious effect on the activity of reverse transcriptase, which confirmed that the lower activity of HIV-1 reverse transcriptase in AAT-pretreated CD4+ T cells was due to the lower amount of HIV-1 reverse transcriptase entry into these cells (Fig. 3b). As the positive control, emtricitabine almost completely blocked the activity of HIV-1 reverse transcriptase without altering the viability of CD4+ T cells (>98 % decrease; *p* < 0.001) (Fig. 3b and Additional file 2: Figure S2). When activated CD4+ T cells were infected with HIV-1_92US714_ or HIV-1_91US054,_ similar results were also obtained (Additional file 5: Figure S5). Thus, these results suggested that AAT inhibited HIV-1 infection by lowering the amount of virus entry into CD4+ T cells.

**Fig. 4:**
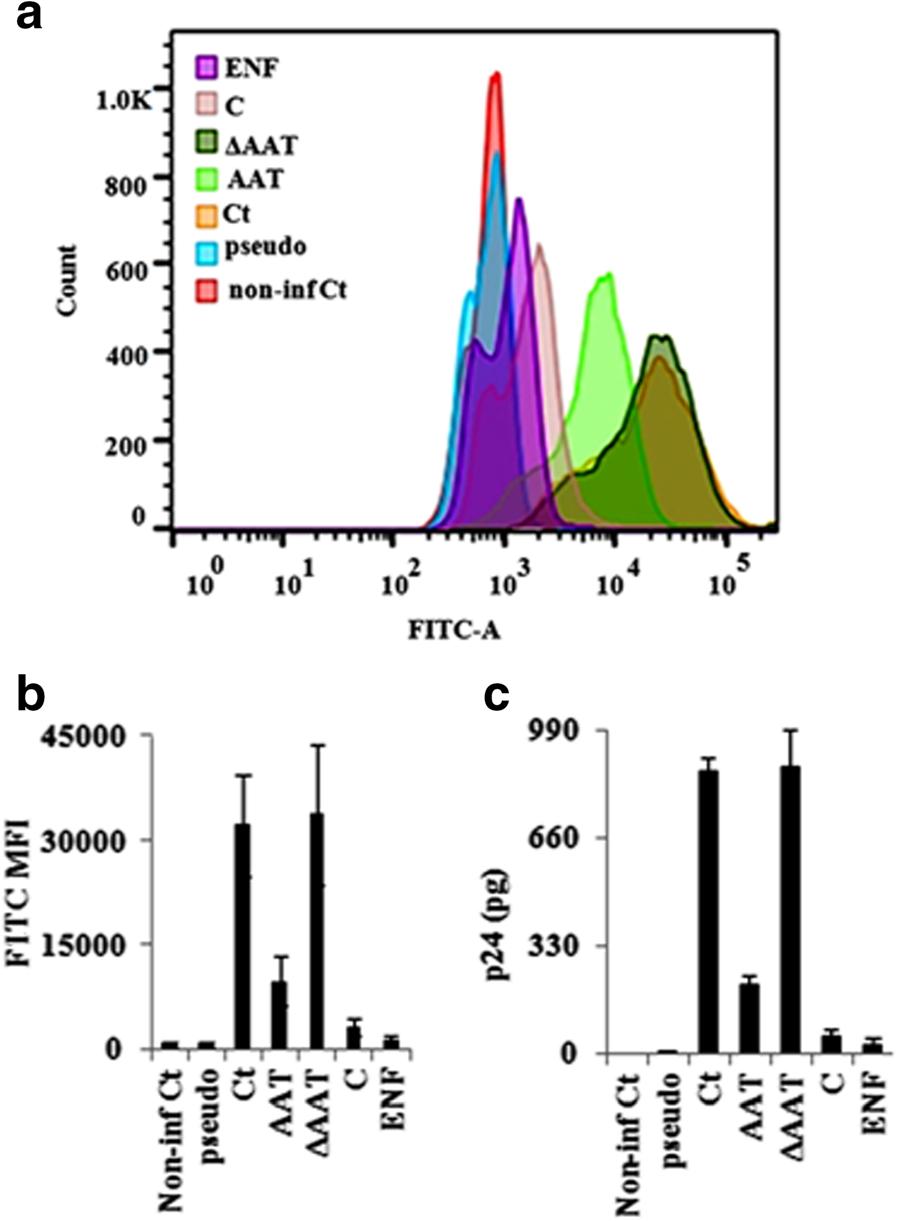
AAT and C inhibited HIV-1 entry in CD4+ T cells. CD4+ T cells were pretreated in the presence or absence of AAT, C, ΔAAT or HIV-1 entry inhibitor enfuvirtide (10^−3^ g/L) and then infected by HIV-1_NL4.3_ (HIV-1 Gag-iGFP) or VSV-G-pseudotyped HIV-1 _NL4.3_ (VSV-G-pseudotyped HIV-1 Gag-iGFP) without removing the reagents. Next, infected CD4+ T cells were collected to detect GFP on flow cytometer. As the negative control, one group of non-infected CD4+ T cells was also analyzed (Non-infec Ct) (**a**). The mean fluorescence intensity of each group was also plotted from three in-dependent experiments on three different donors (**b**). Meanwhile, these CD4+ T cells were also collected to extract whole cell proteins. HIV-1 entry into CD4+ T cells were determined by measuring cytosolic HIV-1 p24 (**c**). Ct: HIV-1 Gag-iGFP-infected CD4+ T cells without reagent treatment. Pseudo: VSV-G-pseudotyped HIV-1 Gag-iGFP-treated CD4+ T cells without reagent treatment; ENF: enfuvirtide treatment; Non-infec Ct: non-infected CD4+ T cells

To confirm that AAT did inhibit HIV-1 entry, HIV-1_NL4.3_ with a GFP insertion within the gag region (HIV-1 Gag-iGFP) was used to carry out the assay [30]. As the control for gp120/gp41 and CD4/co-receptor interaction, HIV-1_NL4.3_ with a GFP insertion within the gag region and VSV-G replacing of Env (VSV-G-pseudotyped HIV-1 Gag-iGFP) was also included. Since HIV-1 fusion with host cell leads to viral core release into the host cell [18], host cells would obtain GFP signal upon HIV-1 Gag-iGFP infection. Meanwhile, host cells could not obtain any GFP signal from VSV-G-pseudotyped HIV-1 Gag-iGFP because VSV-G-pseudotyped HIV-1 Gag-iGFP did not have gp120/gp41 to interact with host cells and the trans-infection of HIV was blocked with the presence of endocytosis inhibitor cocktails in the conditioned culture medium. CD4+ T cells were therefore incubated in the presence or absence of AAT, C, ΔAAT or HIV-1 fusion inhibitor enfuvirtide and then cultured with HIV-1_NL4.3_ Gag-iGFP or VSV-G-pseudotyped HIV-1 Gag-iGFP. After infection, resultant cells were collected to detect HIV-1 entry on flow cytometer (the gate information was showed in Additional file 6: Figure S6). As expected, the results revealed that GFP signal was detected only in CD4+ T cells with HIV-1_NL4.3_ Gag-iGFP treatment, not VSV-G-pseudotyped HIV-1 Gag-iGFP. AAT significantly inhibited HIV-1 entry (>60 % decrease; *p* < 0.005) (Fig. 4a and b). The inhibitory effect of C on HIV-1 entry was stronger than that of AAT (>50 % decrease; *p* < 0.01) (Fig. 4a and b). Meanwhile, as the positive control of HIV-1 entry, enfuvirtide almost completely blocked HIV-1 Gag-iGFP entry without affecting the viability of these T cells (>90 % decrease; *p* < 0.005) (Fig. 4a, b and Additional file 2: Figure S2). Additionally, cytosolic viral p24 was detected in CD4+ T cells with HIV-1_NL4.3_ Gag-iGFP treatment, but not in CD4+ T cells with VSV-G-pseudotyped HIV-1 Gag-iGFP treatment. The amount of cytosolic viral p24 was also lower in AAT-pretreated CD4+ T cells (>60 % decrease; *p* < 0.005) (Fig. 4c). The inhibitory effect of C on HIV-1_NL4.3_ Gag-iGFP entry was also stronger than that of intact AAT (>50 % decrease; *p* < 0.01) (Fig. 4c). Meanwhile, Enfuvirtide treatment completely blocked HIV-1 Gag-iGFP entry into CD4+ T cells (>90 % decrease; *p* < 0.001) (Fig. 4c). Additionally, AAT also inhibited the entry of primary isolate HIV-1_92US714_ and HIV-1_91US054_ into CD4+ T cells (Additional file 7: Figure S7). Collectively, these results confirmed that AAT inhibited HIV-1 infection by blocking virus entry into CD4+ T cells.

**Fig. 5:**
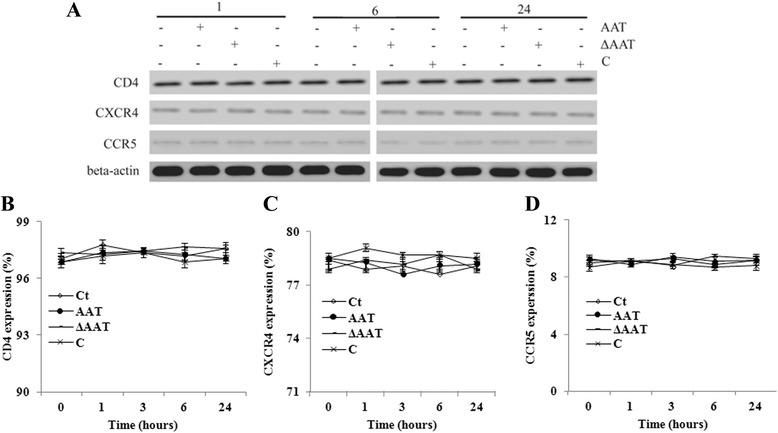
AAT, C and ΔAAT had no obvious effect on CD4, CCR5 and CXCR4 expression on CD4+ T cells. CD4+ T cells were incubated in the presence or absence of AAT/C/ΔAAT and then collected to extract whole cell proteins. The expression of CD4, CCR5 and CXCR4 was detected by Western blot. β-actin was detected as the loading control (**a**). Meanwhile, the membrane level of CD4 (**b**), CCR5 (**c**) and CXCR4 (**d**) was also analyzed on these CD4+ T cells using flow cytometry. Ct: control group without reagent treatment

To inhibit HIV-1 entry, AAT could down-regulate CD4, CXCR4 or CCR5 expression on host T lymphocytes. However, after CD4+ T cells were treated with AAT, C or ΔAAT, the expression of these receptors and co-receptors had no obvious change in the whole cell extract (Fig. 5a). Meanwhile, AAT, C and ΔAAT also had no obvious effect on the expression of these receptors on the plasma membrane (Fig. 5b-d) (the gate information for CD4+ T cells was shown in Additional file 6: Figure S6).

As an alternative mechanism, AAT might directly interact with CD4, CCR5 or CXCR4, or other known HIV-1 infection-related proteins on CD4+ T cells to interfere with the interaction between HIV-1 particles and host cells. To test this postulate, CD4+ T cells were incubated with AAT or ΔAAT to precipitate the cytoplasm membrane proteins interacting with AAT or ΔAAT for protein identification by peptide mass fingerprinting assay. The results revealed that precipitated complexes contained low density lipoprotein receptor-related protein 1, ATP-binding cassette protein and solute carrier protein, which are not related to HIV-1 entry in CD4+ T cells [24]. Meanwhile, CD4, CCR5, CXCR4 and other known HIV-1 infection-related proteins were not detected in precipitated complexes (data not shown).

**Fig. 6:**
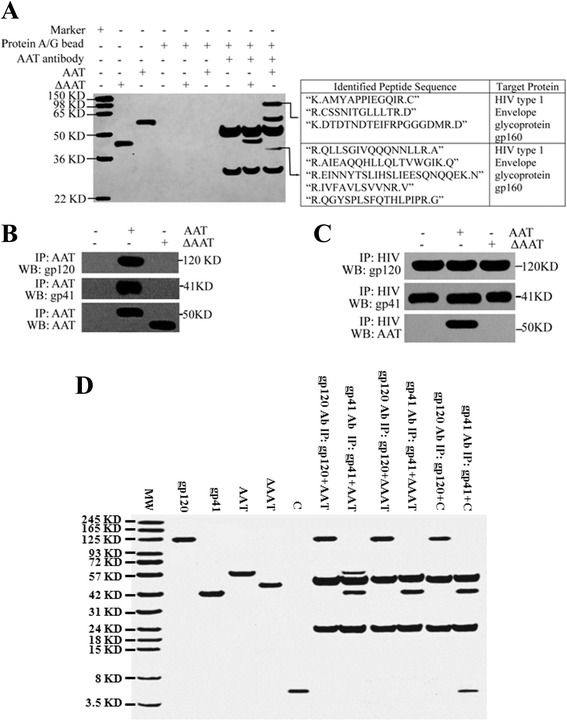
AAT and C directly interacted with gp41. HIV-1_NL4.3_ was incubated in the presence or absence of AAT/ΔAAT and then concentrated by ultracentrifugation (100,000 × g for 2 h at 4 °C) to extract viral proteins. AAT antibody was then added to viral proteins to precipitate proteins interacting with AAT/ΔAAT. Next, each specific protein was identified by peptide mass fingerprinting assay (**a**). Meanwhile, after concentrating, the virus was also divided into two aliquots. One aliquot was lysed to extract whole viral proteins and AAT antibody was then added to precipitate the proteins interacting with AAT/ΔAAT. AAT, gp120 or gp41 in the precipitated proteins were detected by immunoblotting (**b**). The other aliquot was incubated with gp120 antibody to precipitate proteins interacting with viral particles. Subsequently, precipitated viruses were lysed to detect AAT, gp120 and gp41 by Immunoblotting (**c**). Moreover, AAT/C/ΔAAT was also cultured with gp120 or gp41. Next, gp120 or gp41 antibody was added to precipitate. The direct interaction between AAT/C/ΔAAT and gp120/gp41 was determined by separating precipitated proteins with SDS-PAGE and proteins were visualized by Coomassie blue staining (**d**). MW: molecular weight marker; IP: immunoprecipitation; WB: western blotting (immunoblotting)

**Fig. 7:**
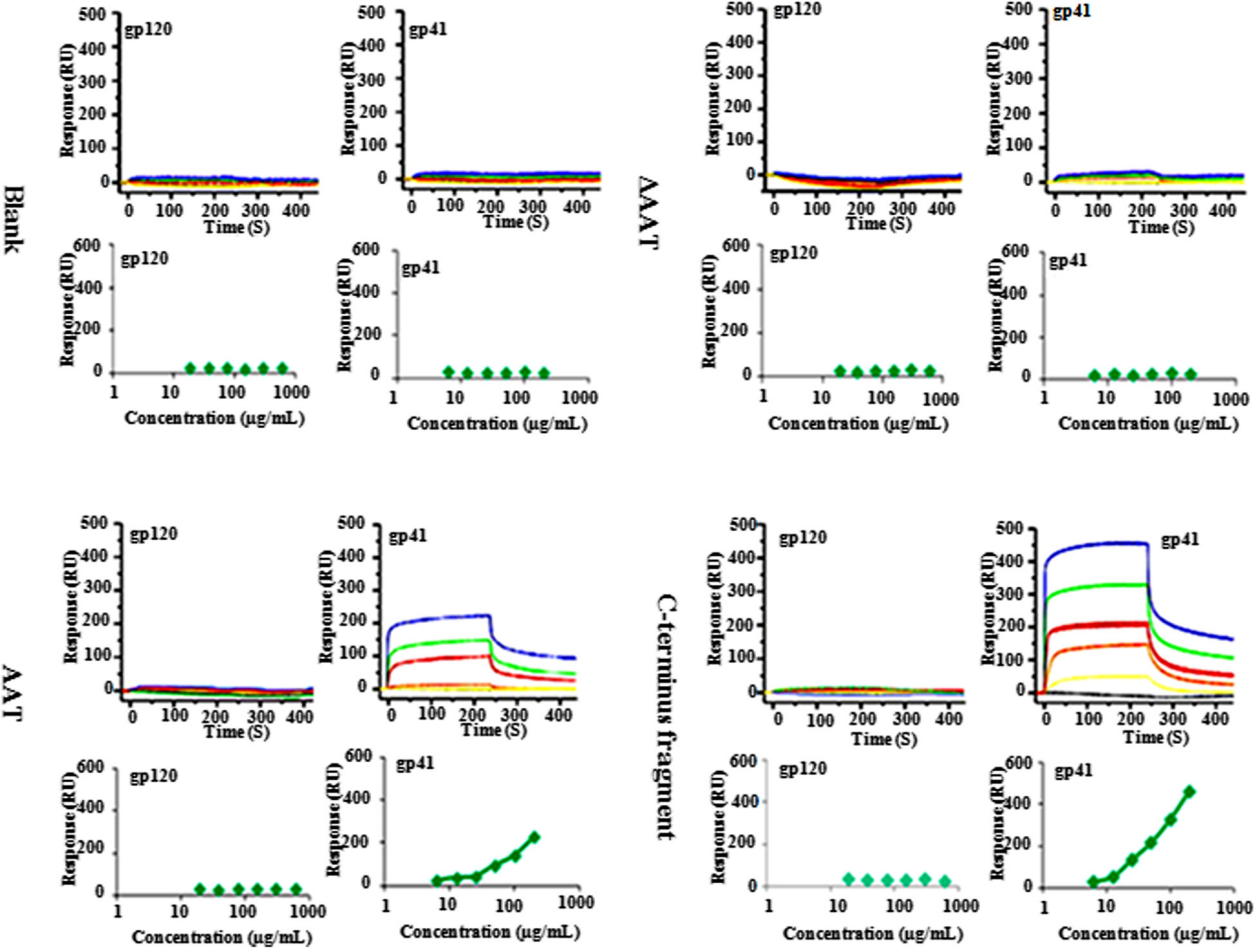
SPR assay of AAT/C and gp41 interaction. gp120 (6.25–200 μg/mL) and gp41 (18.75–600 μg/mL) were analyzed for binding to immobilized AAT, C or ΔAAT. Representative Sensorgrams were displayed and the amount of bound gp120 or gp41 was shown over time. Binding curves revealing saturable kinetics for the interaction were also plotted

As another way to block HIV-1 entry, AAT might directly interact with HIV-1 to block its entry into CD4+ T cells. To test this hypothesis, HIV-1_NL4.3_ was incubated with AAT or ΔAAT. Viral membrane proteins interacting with AAT or ΔAAT were then precipitated and identified by a peptide mass fingerprinting assay. The results revealed that precipitated complexes from the AAT/HIV-1_NL4.3_ co-culture system contained gp120 and gp41. However, no viral protein was detected in the precipitated complexes from the ΔAAT/HIV-1_NL4.3_ co-culture system (Fig. 6a). Moreover, gp120 and gp41 were also detected in precipitated viral proteins from AAT/HIV-1_NL4.3_ by Western blot. However, gp120 and gp41 could not be detected in precipitated viral membrane proteins from ΔAAT/HIV-1_NL4.3_ (Fig. 6b). Furthermore, when HIV-1_NL4.3_ was co-cultured with AAT or ΔAAT to precipitate virus with gp120 antibody, only AAT was found to directly interact with HIV-1 (Fig. 6c). When AAT, C or ΔAAT was co-cultured with gp120 or gp41 to detect the interaction between AAT/C/ΔAAT and gp120/gp41, the results demonstrated that C and AAT could directly interact with gp41 (Fig. 6d). When AAT, C or ΔAAT was immobilized on a carboxymethylated CM5 sensor chip and gp120 or gp41 was then applied to detect the direct interaction by SPR assay, the results revealed that only gp41 interacted with AAT and C (Fig. 7). Therefore, these results together suggested that the C-terminus of AAT, but not other domains, directly interacted with gp41, which might mediate the inhibition of HIV-1 entry.

## Discussion

The study of a clinic case reveals that pre-existing AAT deficiency is associated with accelerated HIV/AIDS progression [13, 18]. Studies reveal that AAT inhibits HIV-1 replication [10, 11, 14, 15]. Constitutive expression of AAT inhibits HIV-1 replication by blocking gp160 and p55 processing in cell lines or primary human lymphocytes [31]. Meanwhile, AAT also inhibits HIV-1 replication by blocking the activation of NF-kB [14, 15, 24, 25, 32]. In the present study, we found that viral replication in AAT-pretreated CD4+ T cells was much lower than that in CD4+ T cells without AAT pretreatment, suggesting that AAT might exert its inhibitory effect on both HIV-1 infection and replication. However, several critical issues still need to be addressed. In the present study, our results show that AAT and C, but not ΔAAT, directly interact with gp41, which might then inhibit HIV-1 entry into CD4+ T cells. Moreover, AAT/ΔAAT/C did not directly interfere with the steps of viral RNA reverse transcription and viral DNA integration. To our surprise, the activity of HIV-1 reverse transcriptase decreased with the elongation of incubation time of the cells. This might be caused by the degradation of HIV-1 reverse transcriptase in the cytosol of CD4+ T cells, which is a general bioprocess in the cell and also leaves us a good topic to follow in the future. Therefore, these observations eliminate the concerns that 52KD AAT might be too big to directly interact with gp120-covered gp41 on the membrane of HIV-1 viral particle. The results also indicate that AAT C-terminus is the only essential functional domain to inhibit HIV-1 infection.

Normally, HIV-1 infection begins with the interaction between gp120/gp41 and CD4/co-receptors, which is then followed by viral core entry, RNA reverse transcription and DNA integration into the host genome [19, 22]. In the present study, AAT, C and ΔAAT did not directly target the steps of viral RNA transcription and viral DNA integration, which means that AAT inhibits HIV-1 infection through blocking virus entry. Although studies demonstrate that AAT inhibits HIV-1 replication in infected lymphocytes [10–15, 31], relatively fewer reports investigate whether AAT inhibits HIV-1 infection of uninfected cells and the mechanism is not fully elucidated yet. Usually, HIV-1 entry into host cells involves the interaction between viral gp120/gp41 and host CD4/CXCR4 or CCR5 [33]. Some researchers, however, suggest that AAT inhibition of HV-1 entry is related to the interaction between AAT and host cell membrane proteins [34]. In the present study, our results did not provide any indication to suggest that AAT interacts with host membrane proteins, thereby inhibiting HIV-1 entry. AAT also did not alter the expression of CD4, CXCR4 and CCR5. Moreover, AAT also did not directly interact with CD4, CCR5, CXCR4 and other HIV-1 infection-related proteins. In contrast, AAT directly interacted with viral gp41. These observations together suggest that intact AAT could interact with viral gp41 to interfere with the interaction between gp120/gp41 and CD4/CCR5 or/CXCR4 and thereby inhibiting HIV-1 entry, which is consistent with previous studies [35]. Moreover, AAT without the C-terminus could not interact with gp41, which indicates that the C-terminus of AAT is an essential functional domain. Munch et al.’s study also reveals that VIRIP at the C-terminus of AAT inhibits HIV-1 infection [12]. When ΔAAT, AAT or synthesized C-terminus fragment of AAT was cultured with gp41 or gp120, we detected direct interaction between AAT/synthesized C-terminus fragment of AAT, but not ΔAAT, and gp41. Thus, collectively with the findings of Munch’s [13], it is clear that the inhibitory effect of AAT on HIV-1 infection is mediated through the direct interaction between the C-terminus of AAT and gp41, which then interferes with the entry of HIV-1 into the host cells.

## Conclusion

Studies have showed that AAT inhibits HIV-1 replication [15, 36]. Our previous study reveals that AAT enters the cytosol of infected CD4+ T cells and directly interacts with cytosolic IkBα to alter its ubiquitinylation pattern. The change of IkBα ubiquitinylation pattern results in the inhibition of NF-kB activation [15, 24]. In infected cells, NF-kB activation is critical for HIV-1 replication [37]. In the present study, our results reveal that AAT inhibits HIV-1 infection not by directly targeting the steps of viral RNA reverse transcription and viral DNA integration. The inhibitory effect of AAT on HIV-1 infection is mediated through the direct interaction between AAT's C-terminus and gp41, which thereby inhibits HIV-1 entry into the host cells. Therefore, these results together indicate that AAT works through multiple ways to exert its inhibitory effects on HIV/AIDS pathogenesis, which may provide useful information for drug development for HIV/AIDS treatment.

## Abbreviations

AAT, alpha-1-antitrypsin; AIDS, acquired immune deficiency syndrome; APC, allophycocyanin; C, synthesized C-terminus of AAT; CM, complete culture medium; DSP, dithiobis succinimidylpropionate; ENF, HIV-1 entry inhibitor enfuvirtide; FITC, isothiocyanate; FTC, HIV-1 reverse transcriptase inhibitor emtricitabine; HIV-1, human immunodeficiency virus type 1; HPLC-MS, high-performance liquid chromatography-mass spectrometry system; IkBα, nuclear factor kB inhibitor; NF-kB, nuclear factor kB; RAL, HIV-1 integrase inhibitor raltegravir; SPR, surface plasmon resonance; ΔAAT, C terminus-truncated AAT

## Additional files

Additional file 1: Figure S1. Purity and quality of AAT, ΔAAT and C. ΔAAT was prepared following the protocol in Materials and. Next, AAT, purified ΔAAT and C were analyzed on 4–20 % Tris-Glycin SDS-PAGE gel and analyzed by in-gel digestion mass fingerprinting assay (*A*). Aligned sequences were also shown for AAT, ΔAAT and C (*B*). (TIF 6959 kb) Additional file 2: Figure S2. AAT, raltegravir, emtricitabine and enfuvirtide had no obvious effect on the viability of CD4+ T cells. CD4+ T cells were pretreated with or without AAT for 1 h. Next, these cells were incubated with AAT, raltegravir (10^−4^ g/L), emtricitabine (10^−3^ g/L), or enfuvirtide (10^−3^ g/L) for indicated time period. At the end of incubation, cells were collected to detect the viability using trypan blue staining. RAL: raltegravir treatment; FTC: emtricitabine treatment; ENF: enfuvirtide treatment. (TIF 975 kb) Additional file 3: Figure S3. 0.5 mg/mL AAT did not induce the precipitation of HIV-1NL4.3 particles. Same amount of HIV-1NL4.3 viral stock was incubated with the presence or absence of 0.5 mg/mL AAT (final concentration) and activated CD8+ T cells (10 million in 1 mL; the same concentration as CD4+ T cells to work as a cell loading control). The final concentration and volume for HIV-1NL4.3, AAT and CD8+ T cells were adjusted to be the same as CD4+ T cells infection system. After 2 h’ incubation with gentle shaking at 37° (same as CD4+ T cells infection procedure), the mixtures were centrifuged for 15 min at 10,000 × g to pellet down any possible precipitation. The supernatant was collected to detect HIV-1 p24 (A) and viral RNA (B). (TIF 1557 kb) Additional file 4: Figure S4. AAT, C and ΔAAT did not directly target the integration of primary HIV-1 DNA into the host genome. CD4+ T were incubated in the presence or absence of AAT/C/ΔAAT and then infected by HIV-1_91US054_ (*A*) or HIV-1_92US714_ (*C*) without removing reagents. Next, infected CD4+ T cells were washed to remove unbound viruses and reagents and incubated for 1, 6, 12, 18, 24, or 30 h in the presence or absence of AAT/C/ΔAAT (same condition as before infection) to isolate DNA. The integration of HIV-1 viral DNA was detected by Alu-PCR (*A* and *C*). Additionally, CD4+ T cells were also infected with HIV-1_91US054_ (*B*) or HIV-1_92US714_ (*D*) without AAT/C/ΔAAT pretreatment and then incubated with the presence or absence of AAT/C/ΔAAT. After 0, 5, 11, 17, 23, or 29 h’ incubation, DNA was extracted to detect viral DNA integration (*B* and *D*). Genomic beta-globin was also detected as an endogenous control. (TIF 1195 kb) Additional file 5: Figure S5. AAT, C and ΔAAT did not directly affect the activity of HIV-1 reverse transcriptase. CD4+ T cells were pretreated in the presence or absence of AAT/C/ΔAAT and then infected by HIV-1_91US054_ (*A*) or HIV-1_92US714_ (*C*) without removing the reagents. Infected CD4+ T cells were then washed three times to remove unbound viruses and incubated in the presence or absence of AAT/C/ΔAAT (same condition as before infection) for 1, 6, 12, 18, 24, or 30 h to isolate whole cell and viral proteins. HIV-1 reverse transcriptase activity was detected. Meanwhile, CD4+ T cells were also infected with primary HIV-1_91US054_ (*B*) or HIV-1_92US714_ (*D*) without AAT/C/ΔAAT pretreatment and then incubated in the presence or absence of AAT/C/ΔAAT. After 0, 5, 11, 17, 23, or 29 h incubation, whole cell proteins with viral proteins were extracted from these CD4+ T cells to detect the activity of HIV-1 reverse transcriptase. (TIF 1480 kb) Additional file 6: Figure S6. Flow cytometry gate information for CD4+ T cell selection and CD4/CCR5/CXCR4 detection on selected CD4+ T cells. (TIF 8012 kb) Additional file 7: Figure S7. AAT and C inhibited HIV-1 entry. CD4+ T cells were treated in the presence or absence of AAT/C/ΔAAT and then infected by HIV-1_91US054_ (*A*) or HIV-1_92US714_ (*B*) without removing the reagents. Next, infected CD4+ T cells were collected to extract whole cell proteins (including p24). HIV-1 entry was determined by measuring cytosolic p24. (TIF 327 kb)
