# Supplementary figures and images for: Alpha-1-antitrypsin interacts with gp41 to block HIV-1 entry into CD4+ T lymphocytes
Source: BMC Microbiol. 2016 Jul 29;16:172. doi: 10.1186/s12866-016-0751-2 (PMC4966588; doi:10.1186/s12866-016-0751-2)

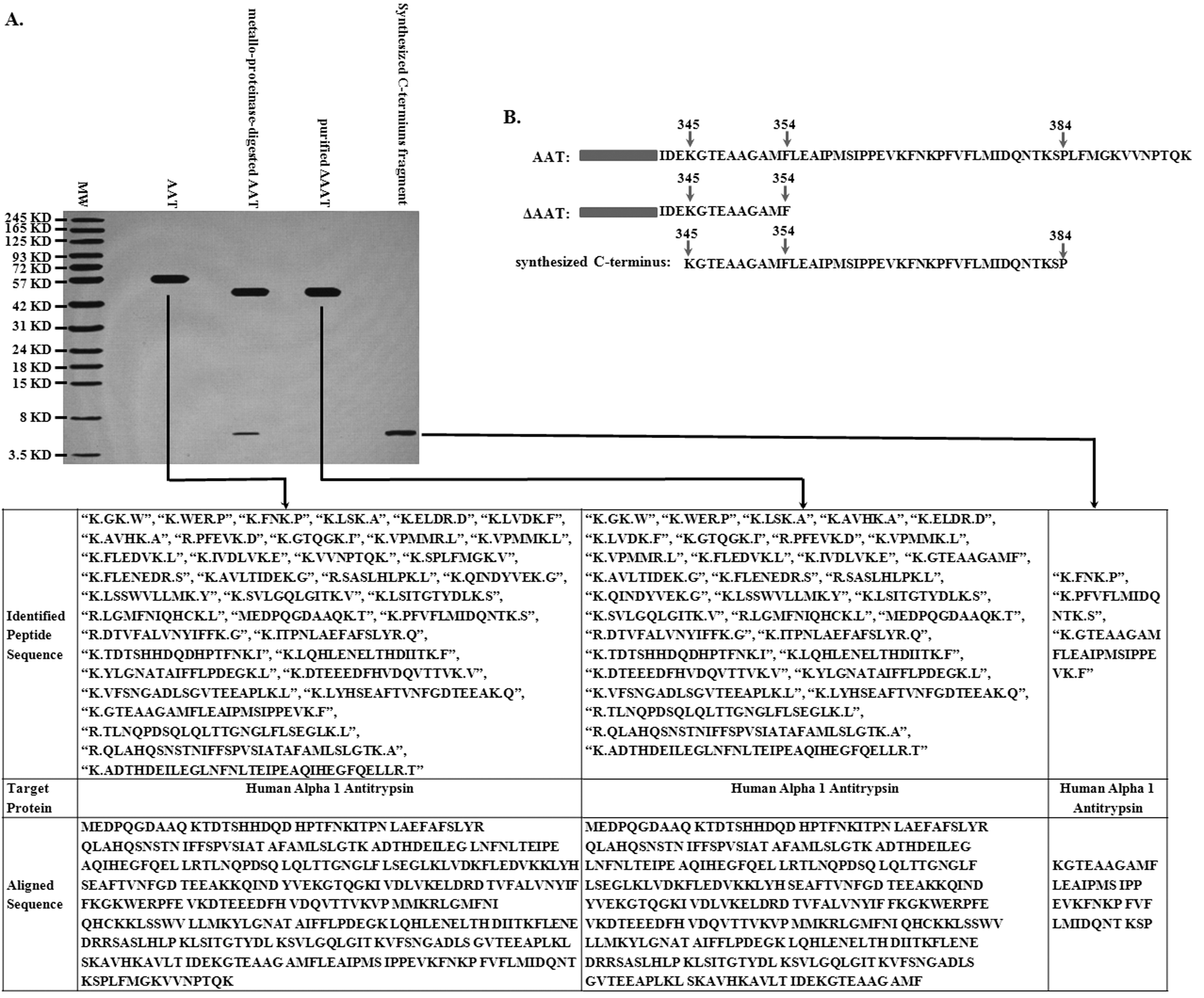

Supplement: Additional file 1: Figure S1. — Purity and quality of AAT, ΔAAT and C. ΔAAT was prepared following the protocol in Materials and. Next, AAT, purified ΔAAT and C were analyzed on 4–20 % Tris-Glycin SDS-PAGE gel and analyzed by in-gel digestion mass fingerprinting assay (A). Aligned sequences were also shown for AAT, ΔAAT and C (B). (TIF 6959 kb) [file 12866_2016_751_MOESM1_ESM.tif]

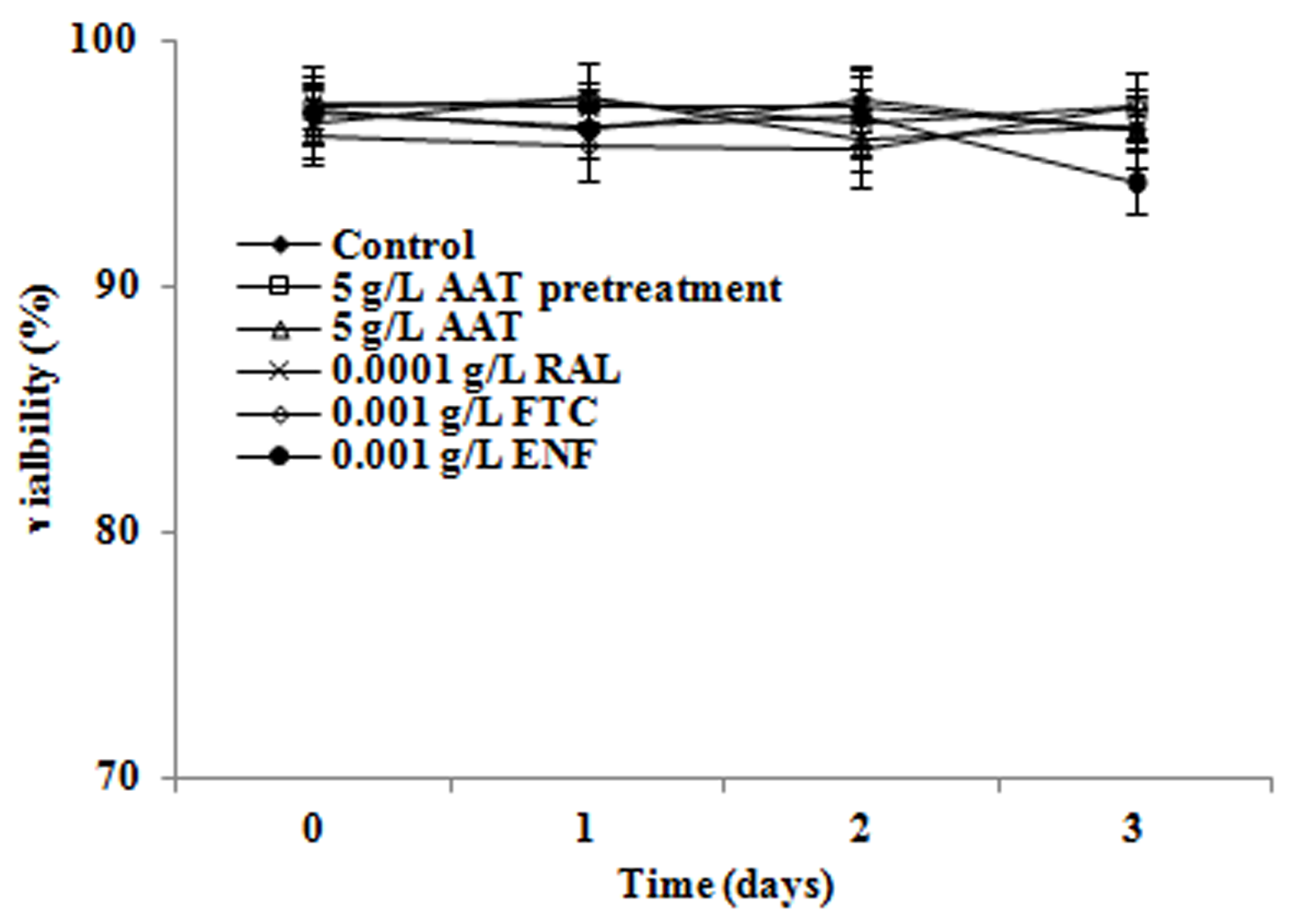

Supplement: Additional file 2: Figure S2. — AAT, raltegravir, emtricitabine and enfuvirtide had no obvious effect on the viability of CD4+ T cells. CD4+ T cells were pretreated with or without AAT for 1 h. Next, these cells were incubated with AAT, raltegravir (10−4 g/L), emtricitabine (10−3 g/L), or enfuvirtide (10−3 g/L) for indicated time period. At the end of incubation, cells were collected to detect the viability using trypan blue staining. RAL: raltegravir treatment; FTC: emtricitabine treatment; ENF: enfuvirtide treatment. (TIF 975 kb) [file 12866_2016_751_MOESM2_ESM.tif]

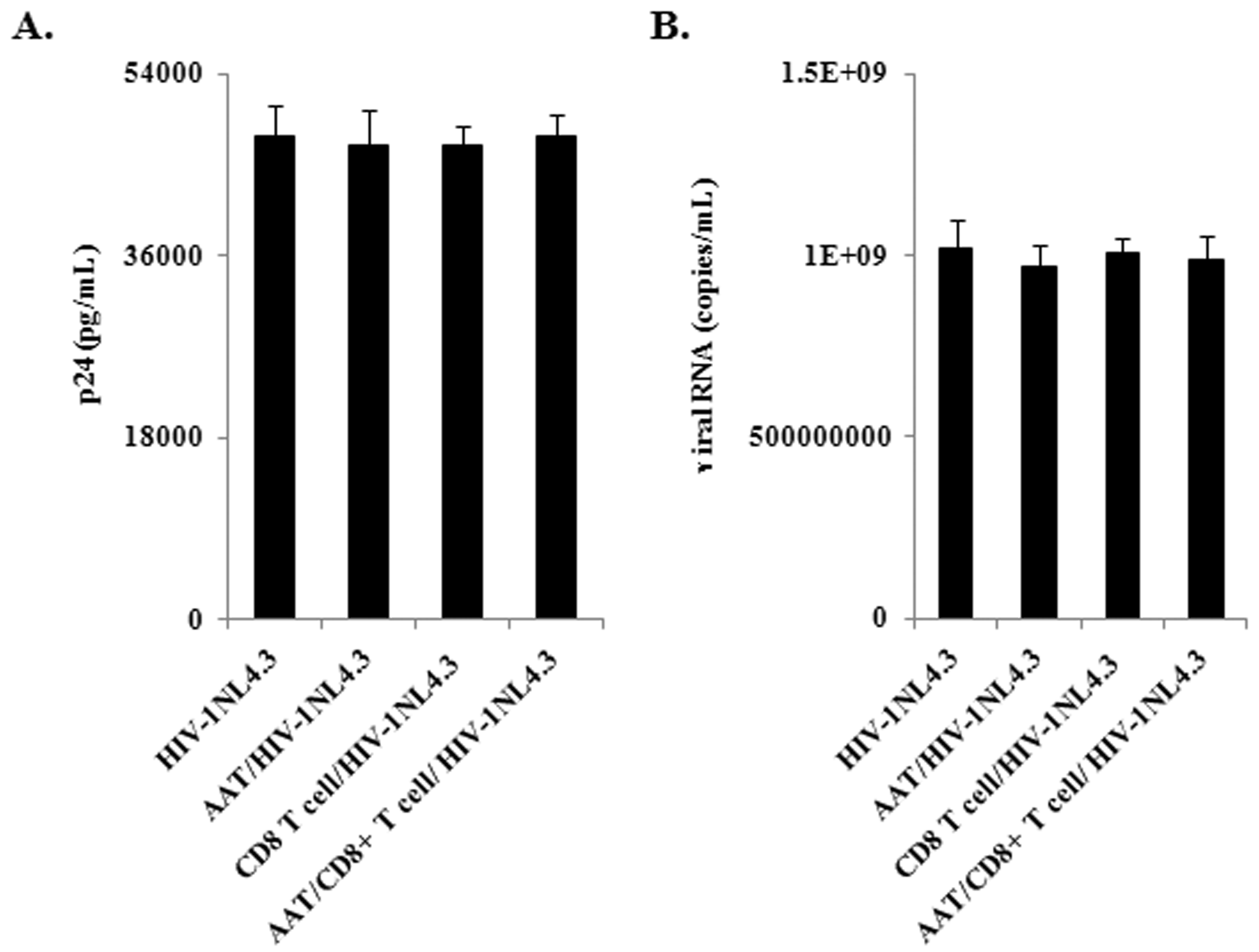

Supplement: Additional file 3: Figure S3. — 0.5 mg/mL AAT did not induce the precipitation of HIV-1NL4.3 particles. Same amount of HIV-1NL4.3 viral stock was incubated with the presence or absence of 0.5 mg/mL AAT (final concentration) and activated CD8+ T cells (10 million in 1 mL; the same concentration as CD4+ T cells to work as a cell loading control). The final concentration and volume for HIV-1NL4.3, AAT and CD8+ T cells were adjusted to be the same as CD4+ T cells infection system. After 2 h’ incubation with gentle shaking at 37° (same as CD4+ T cells infection procedure), the mixtures were centrifuged for 15 min at 10,000 × g to pellet down any possible precipitation. The supernatant was collected to detect HIV-1 p24 (A) and viral RNA (B). (TIF 1557 kb) [file 12866_2016_751_MOESM3_ESM.tif]

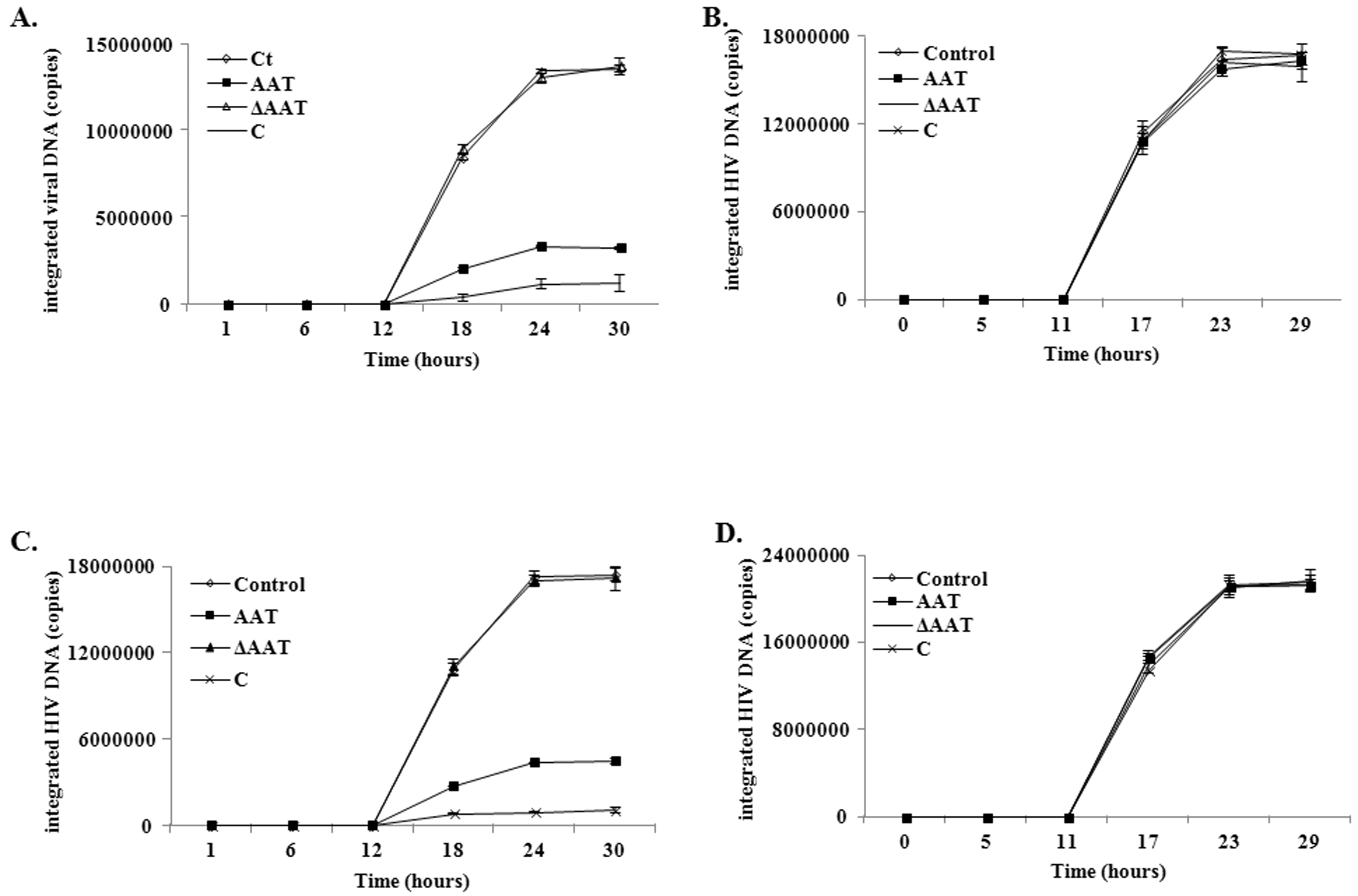

Supplement: Additional file 4: Figure S4. — AAT, C and ΔAAT did not directly target the integration of primary HIV-1 DNA into the host genome. CD4+ T were incubated in the presence or absence of AAT/C/ΔAAT and then infected by HIV-191US054 (A) or HIV-192US714 (C) without removing reagents. Next, infected CD4+ T cells were washed to remove unbound viruses and reagents and incubated for 1, 6, 12, 18, 24, or 30 h in the presence or absence of AAT/C/ΔAAT (same condition as before infection) to isolate DNA. The integration of HIV-1 viral DNA was detected by Alu-PCR (A and C). Additionally, CD4+ T cells were also infected with HIV-191US054 (B) or HIV-192US714 (D) without AAT/C/ΔAAT pretreatment and then incubated with the presence or absence of AAT/C/ΔAAT. After 0, 5, 11, 17, 23, or 29 h’ incubation, DNA was extracted to detect viral DNA integration (B and D). Genomic beta-globin was also detected as an endogenous control. (TIF 1195 kb) [file 12866_2016_751_MOESM4_ESM.tif]

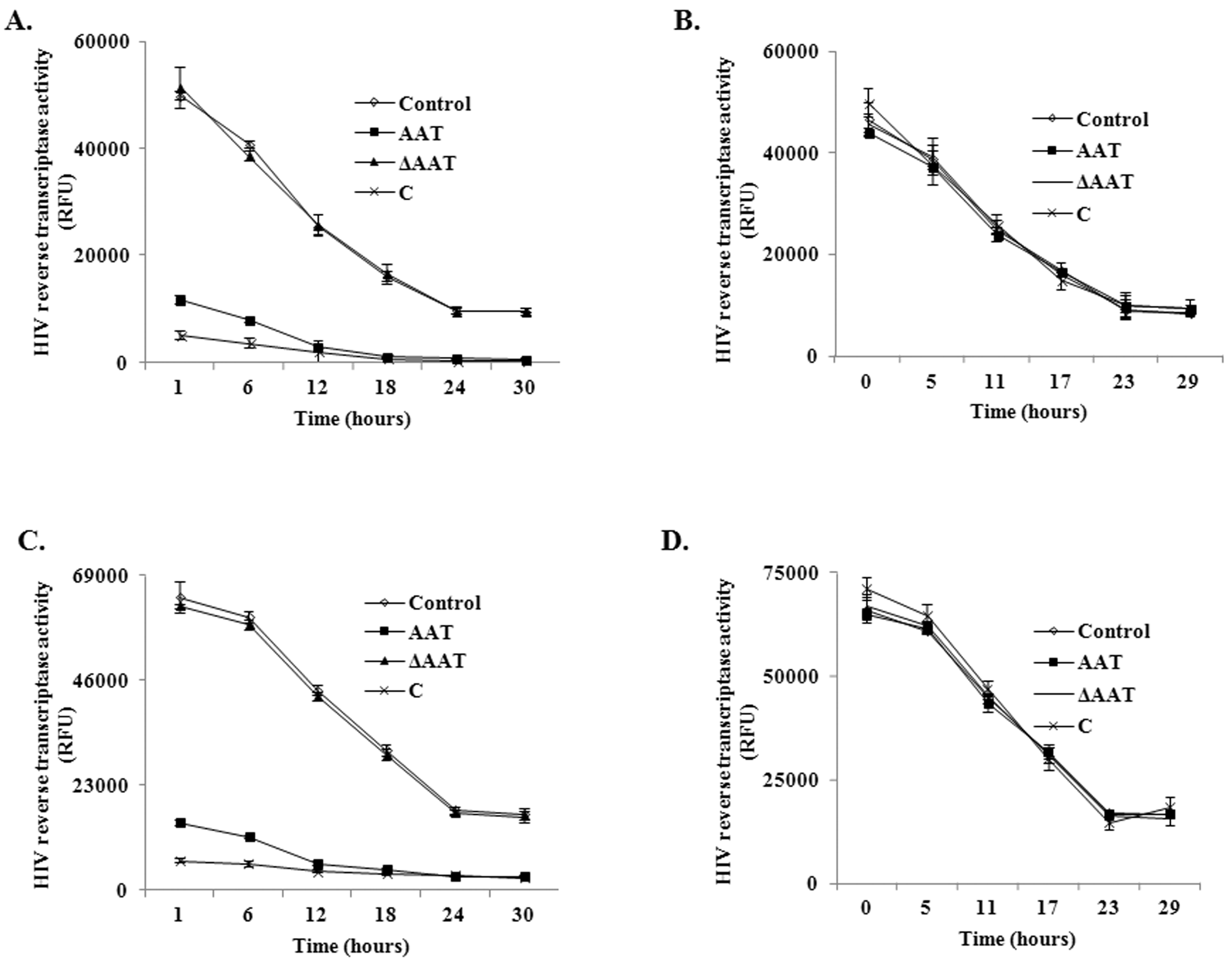

Supplement: Additional file 5: Figure S5. — AAT, C and ΔAAT did not directly affect the activity of HIV-1 reverse transcriptase. CD4+ T cells were pretreated in the presence or absence of AAT/C/ΔAAT and then infected by HIV-191US054 (A) or HIV-192US714 (C) without removing the reagents. Infected CD4+ T cells were then washed three times to remove unbound viruses and incubated in the presence or absence of AAT/C/ΔAAT (same condition as before infection) for 1, 6, 12, 18, 24, or 30 h to isolate whole cell and viral proteins. HIV-1 reverse transcriptase activity was detected. Meanwhile, CD4+ T cells were also infected with primary HIV-191US054 (B) or HIV-192US714 (D) without AAT/C/ΔAAT pretreatment and then incubated in the presence or absence of AAT/C/ΔAAT. After 0, 5, 11, 17, 23, or 29 h incubation, whole cell proteins with viral proteins were extracted from these CD4+ T cells to detect the activity of HIV-1 reverse transcriptase. (TIF 1480 kb) [file 12866_2016_751_MOESM5_ESM.tif]

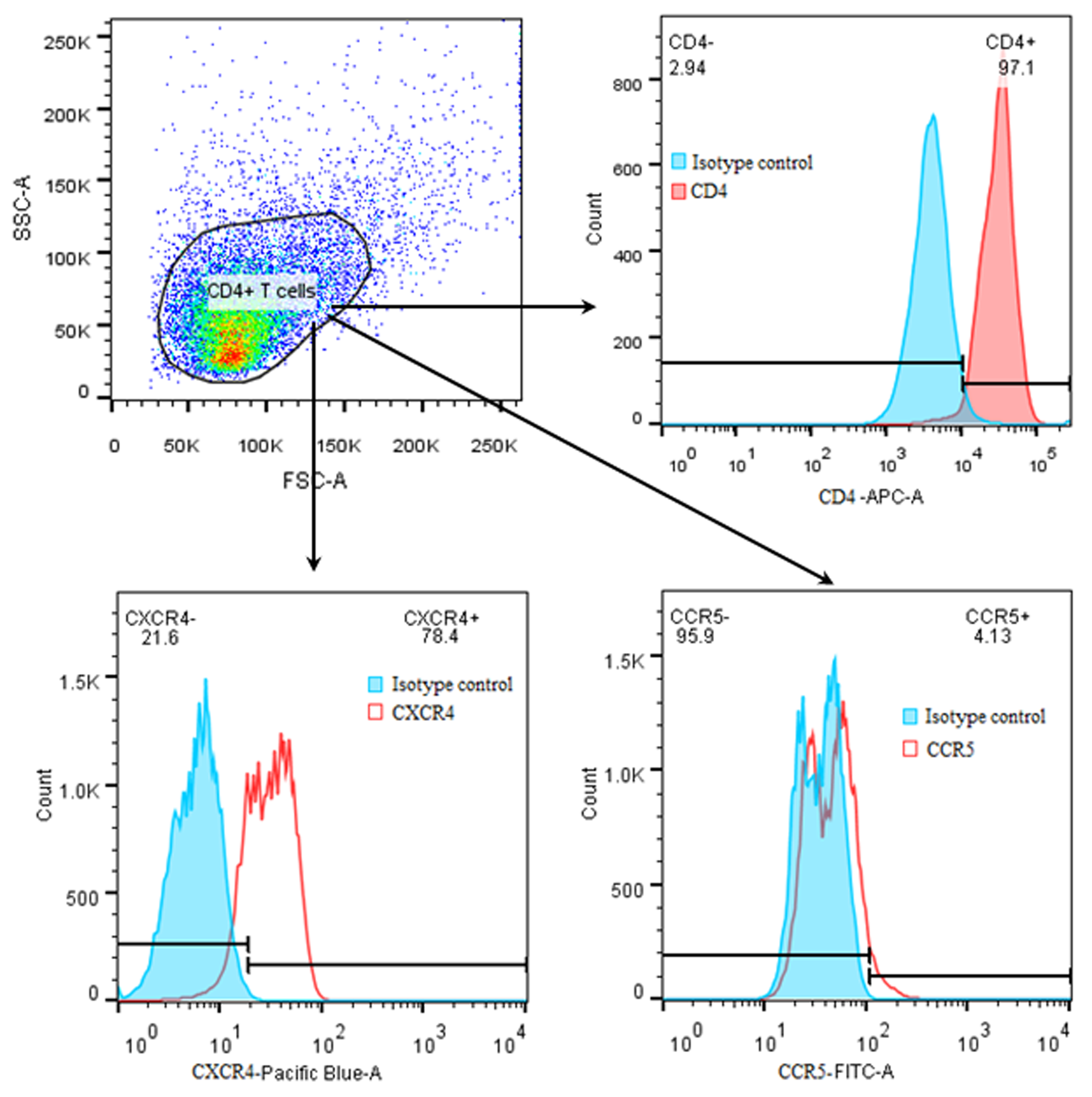

Supplement: Additional file 6: Figure S6. — Flow cytometry gate information for CD4+ T cell selection and CD4/CCR5/CXCR4 detection on selected CD4+ T cells. (TIF 8012 kb) [file 12866_2016_751_MOESM6_ESM.tif]

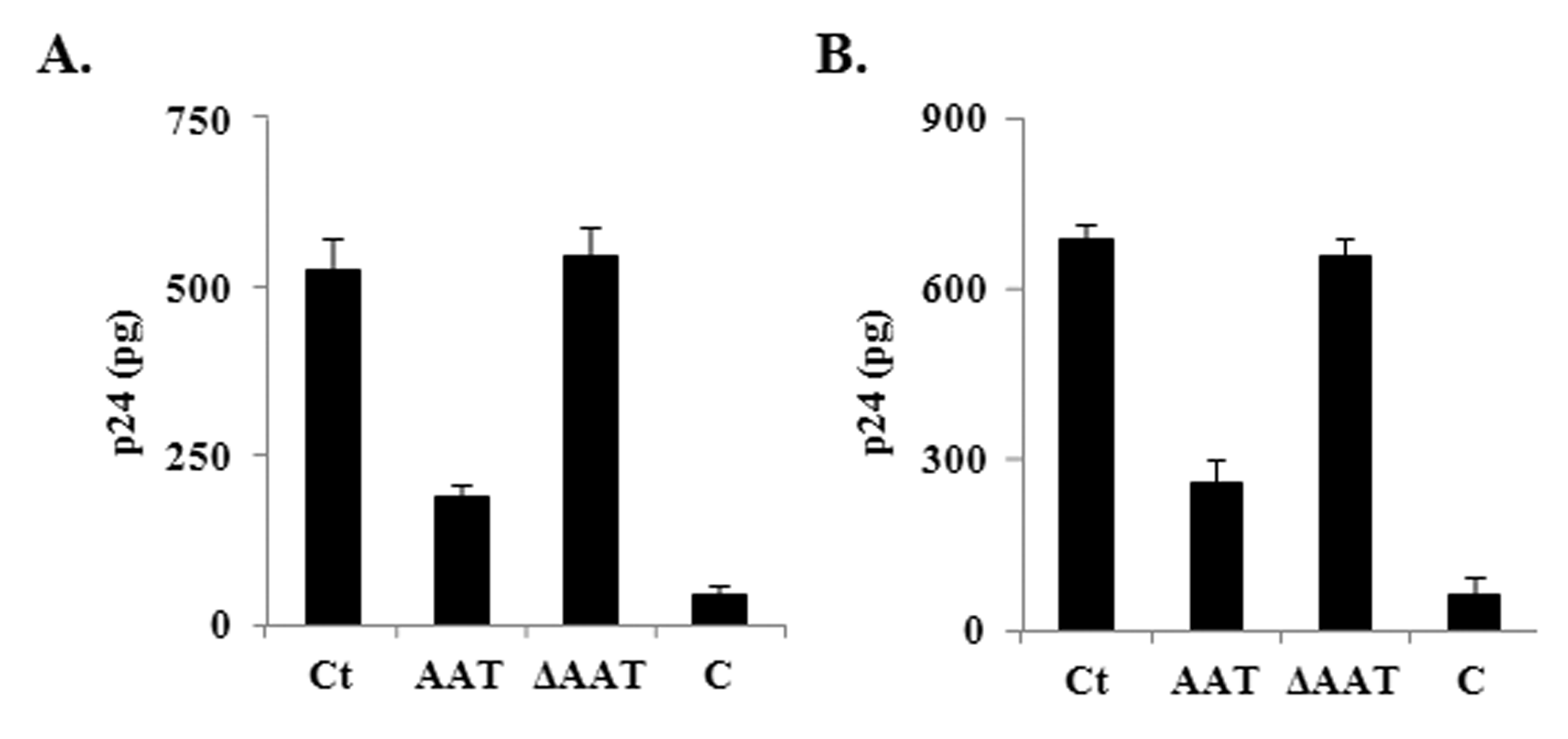

Supplement: Additional file 7: Figure S7. — AAT and C inhibited HIV-1 entry. CD4+ T cells were treated in the presence or absence of AAT/C/ΔAAT and then infected by HIV-191US054 (A) or HIV-192US714 (B) without removing the reagents. Next, infected CD4+ T cells were collected to extract whole cell proteins (including p24). HIV-1 entry was determined by measuring cytosolic p24. (TIF 327 kb) [file 12866_2016_751_MOESM7_ESM.tif]
